# Who or what is to blame? Examining sociodemographic relationships to beliefs about causes, control, and responsibility for cancer and chronic disease prevention in Alberta, Canada

**DOI:** 10.1186/s12889-021-11065-4

**Published:** 2021-06-02

**Authors:** Kimberley D. Curtin, Mathew Thomson, Candace I. J. Nykiforuk

**Affiliations:** Policy, Location and Access in Community Environments (PLACE) Research Lab, School of Public Health, Edmonton Clinic Health Academy, University of Alberta, 11405 - 87 Ave, Edmonton, AB T6G 1C9 Canada

**Keywords:** Chronic disease prevention, Healthy policy, Etiology, Cancer, Attribution theory, Multinomial logistic regression

## Abstract

**Background:**

Beliefs about causes and responsibility for chronic diseases can affect personal behaviour and support for healthy policies. In this research we examined relationships between socio-demographics (sex, age, education, employment, political alignment, perceived health, household income, household size) and perceptions of causes and responsibility for health behaviour, chronic disease correlates, and attitudes about cancer prevention and causes.

**Methods:**

Using data from the 2016 Chronic Disease Prevention survey in which participants (*N* = 1200) from Alberta, Canada responded to items regarding how much they believed personal health behaviours, prevention beliefs, and environmental factors (i.e., healthy eating, physical activity, alcohol, smoking, and where a person lives or works) are linked to getting cancer. Participants also responded to questions about causes and responsibility for obesity, alcohol, and tobacco (i.e., individual or societal). Relationships were examined using multinomial logistic regression on socio-demographics and survey items of interest.

**Results:**

Men (compared to women) were less likely to link regular exercise, or drinking excessive alcohol, to reducing or increasing cancer risk. Similarly, men were less likely to link environmental factors to cancer risk, and more likely to agree that cancer was not preventable, and that treatment is more important than prevention. Finally, men were more likely to believe that alcohol problems are an individual’s fault. Left and central voters were more likely to believe that society was responsible for addressing alcohol, tobacco, and obesity problems compared to right voters. Those with less than post-secondary education were less likely to believe that regular exercise, maintaining a healthy body weight, or eating sufficient fruits and vegetables were linked to cancer - or that society should address obesity - compared to those with more education. Households making above the median income (versus below) were more likely to link a balanced diet with cancer and were less likely to think that tobacco problems were caused by external circumstances.

**Conclusions:**

These results provide insight into the importance of health literacy, message framing, and how socio-demographic factors may impact healthy policy. Men, those with less education, and those with less income are important target groups when promoting health literacy and chronic disease prevention initiatives.

**Supplementary Information:**

The online version contains supplementary material available at 10.1186/s12889-021-11065-4.

## Background

The World Health Organization has highly prioritized the prevention of non-communicable (or chronic) diseases (NCDs) such as heart disease, stroke, cancer, and diabetes, calling them an “invisible epidemic” [1]. There are several modifiable risk factors for chronic diseases including tobacco use, harmful use of alcohol, unhealthy diet, insufficient physical activity, and overweight/obesity. Upstream policy interventions that shape physical and policy environments effectively support improved health at a population level, for example through equitable access and availability of services, supports, and resources [2]. Upstream population interventions can also target the social determinants of health, addressing “the conditions in which people are born, grow, live, work, and age” [3] and the consequential impacts of those conditions on people’s health.

There are numerous interventions that could target social determinants of health, but those focused on individual behaviours are dominant in Western intervention narratives, bolstered by fears of behaviour regulation and nanny-stateism [4, 5]. The nanny metaphor associates an overbearing, intrusiveness to government action in caring for their “infantile” citizens [4]. Focusing on an individual’s responsibility for their health can be seen as ‘strengthening’ rather than ‘blaming’ because it grants personal freedom, control, and autonomy [5], however, the focus on individual behaviour, despite structural barriers to behaviour change, has implied blame on those with chronic diseases [6]. Thus, how a person views the causes and responsibility for chronic diseases can impact personal behaviour choices as well as their support for upstream policies that shape healthy environments [7, 8]. Yet, current empirical literature has seldom investigated how personal characteristics are related to the attribution of causes and responsibility for chronic disease correlates in the context of their support for intervention options ranging from those focused on individual behaviour change to upstream policy interventions. This paper helps to address that gap.

Attribution theory suggests that people assign control and, therefore, responsibility for health conditions to either internal or external forces in order to make sense of them [9]. In this theory, responsibility is related to perceptions of a person’s intention, freedom, and free will. The perceived causes of a person’s condition (e.g., behavioural or circumstantial) determine how much responsibility that person has for their condition, the consequential perceptions, and whether they are offered help [8, 9]. The stigma associated with a condition may also influence perceptions of personal or internal responsibility. For instance, stigmatized persons with perceived behavioural or psychological ‘problems’ (e.g., people with AIDS, people with a substance use disorder, people with obesity) were held responsible for their condition, but those perceived to have uncontrollable ‘problems’ (e.g., cancer, heart disease, paraplegia) were generally not held responsible [10], despite the incontrovertible societal influences shaping many of these ‘problems’.

Causal beliefs about chronic disease also implicate the social determinants of health and upstream policies that impact health. In a large sample of US adults, those who recognized the role of social determinants of health (i.e., health care, genetic, social, and environmental factors), and equated social and health policy, were more likely to be older, women, non-white, liberal, to have less education, lower income, and fair or poor health [11]. Linking socio-demographics to causal beliefs, previous research found that women were more likely to support more intrusive policy interventions to change tobacco and alcohol use, diet, and physical activity [12]. Generally, those who are politically conservative endorse individual responsibility for health and support policies that are largely individual such as nutrition labelling, and education [7, 13]. More left-wing or liberal political ideologies are associated with beliefs in prevention and external environmental and sociological causes and cures for illness [14] and may include junk food and gasoline taxes, advertising bans, and city planning to promote better nutrition and physical activity [13]. Understanding the relationships between these socio-demographic factors and perceptions of chronic disease causation and responsibility can rectify critical knowledge gaps and inform policy and practice interventions targeting those gaps.

Recognizing the need to understand how personal characteristics are related to the attribution of causes and responsibility for chronic disease in the context of support for a range of chronic disease prevention intervention options, the present study examined the relationships between socio-demographic variables (i.e., sex, age, education, employment, federal and provincial political alignment, perceived health, household income, number of people in a household) and perceptions of causes and responsibility for health behaviour, chronic disease correlates, and attitudes about cancer prevention and causes. These variables can provide insight on appetite for policy intervention, and targeted intervention on chronic disease prevention. This research extends previous work by identifying patterns in specific socio-demographic groups, facilitating targeted communication strategies rooted in theory. The objectives of this research were to:

1. Examine how socio-demographic factors are related to Alberta residents’ knowledge and beliefs about individual and environmental links to cancer and cancer prevention.
2. Identify how socio-demographic factors are related to Alberta residents’ perceived causes and responsibility for alcohol use, tobacco use, and obesity.

We hypothesized that women compared to men, and those who were older compared to younger would recognize environmental factors related to cancer, and social responsibility for alcohol use, tobacco use, and obesity [11, 12, 15]. Those who were more politically liberal were expected to recognize environmental causes of cancer and societal responsibility for alcohol, tobacco, and obesity [7, 13, 14]. Those with more education were hypothesized to recognize links between behavioural correlates for chronic disease, but education may be negatively related to awareness of environmental causes of cancer [11]. Those with lower income and poorer health were hypothesized to recognize environmental relationships to cancer and be more likely to recognize social causes of health and health behaviour [11].

## Methods

This manuscript describes a secondary analysis of questions from the 2016 wave of the Chronic Disease Prevention (CDP) survey regarding the knowledge, attitudes, and beliefs of Alberta residents as they pertain to cancer causes and prevention, and the perceived etiology of chronic disease. The CDP survey has been administered in 6 waves since 2009 [16]. Responses were collected through May and June of 2016, with the purpose of this survey being to understand the knowledge, attitudes, and beliefs of policy influencers and the general public on healthy public policy for population-level chronic disease prevention specific to four major risk behaviours: alcohol consumption, unhealthy eating, physical inactivity, and tobacco use. Only data from the general public survey were used for this analysis.

### Participants

Participants (*N* = 1200) from the general public residing in Alberta were sampled via telephone by a professional polling firm using random digit dialing. Respondents were eligible if they were over 18 years of age and resided in Alberta. This procedure used a stratified sampling protocol with targeted samples, stratifying by sex, urban versus rural geography, and age. The sample size of 1200 for the Alberta general public was decided on in order to be representative, with a priori analysis indicating a sample size of 1200 for the province (with 400 each for Calgary CMA, Edmonton CMA, and the rest of the province) produces a two-sided 95% confidence interval with a width equal to 0.058 when the sample proportion is 0.500. The response rate was 8% based on completed interviews and those who were eligible but did not complete the interview, however target sample sizes by strata were achieved. The study protocol was approved by the University of Alberta Research Ethics Board. Table 1 describes participant characteristics of the non-imputed dataset.

**Table 1:** Socio-demographic characteristics of the sample

Demographic	Overall (***N*** = 1200)
Age (mean years [SD])^a^	53.12 (16.04)
Age categories (%)	
18–29 years	104 (8.8)
30–39 years	154 (13.0)
40–49 years	205 (17.3)
50–59 years	270 (22.7)
60–69 years	272 (22.9)
70+ years	182 (15.3)
Sex (%)^e^	
Woman	609 (50.8)
Man	591 (49.3)
Education (%)^b^	
Up to Post-Secondary Education	472 (39.7)
Post-Secondary Graduate	716 (60.3)
Employment (%)^c^	
Full Time	486 (40.6)
Other than Full Time	711 (59.4)
Federal Vote (%)^d^	
Right	454 (56.4)
Centre	275 (34.2)
Left	76 (9.4)
Provincial Vote (%)^i^	
Right	510 (60.4)
Centre	105 (12.4)
Left	230 (27.2)
Health (%)^f^	
Good or Better	1024 (85.4)
Fair or Worse	175 (14.6)
Household Income (%)^g^	
Below Median (<$70,000)	383 (36.6)
Above Median (≥$70,000)	663 (63.4)
Household Size (%)^h^	
Single Person Household	233 (19.5)
Two Person Household	442 (37.0)
Three Person Household	187 (15.7)
Four Plus Person Household	332 (27.8)
Population Centre (%)^e^	
Outside	400 (33.3)
Inside	800 (66.7)

Note: Total percent may not sum to 100 due to rounding

^a^ missingness: *n* = 13 (1.1%)

^b^ missingness: *n* = 12 (1.0%)

^c^ missingness: *n* = 3 (0.3%)

^d^ missingness: *n* = 395 (32.9%)

^e^ missingness: *n* = 0 (0%)

^f^ missingness: *n* = 1 (0.1%)

^g^ missingness: *n* = 154 (12.8%)

^h^ missingness: *n* = 6 (0.5%)

^i^ missingness: *n* = 355 (29.6%)

### Measures

Survey items were generated from validated instruments used in research on tobacco control and alcohol policy [17–19]. The subscales were validated using factor analyses and the results are reported in Nykiforuk et al. [20].

#### Cancer causes and prevention

Responses to the stem, “Please indicate how much you think each of the following items is linked to a person’s chances of getting cancer” were rated on a four-point Likert-style scale from “Definitely Linked” to “Definitely is Not Linked” (1 = “Definitely Linked”, 2 = ‘Might be Linked”, 3 = “Probably is Not Linked”, and 4 = “Definitely is Not Linked”). These included individual factors (e.g., regular exercise, smoking cigarettes, drinking excessive alcohol, eating a balanced diet), and environmental exposures (i.e., residing near industrial facilities, where someone goes to school, and where a person lives). Additionally, respondents ranked their agreement with a series of statements intended to capture their beliefs about cancer prevention on a four-point Likert-style scale (1 = “Strongly Agree”, 2 = “Agree”, 3 = “Disagree”, and 4 = “Strongly Disagree”). Statements included “Most cancers are preventable” and “Getting cancer is just bad luck, since there is nothing people can do about it”. Responses to these questions were then dichotomized into Linked and Not Linked, or Agree and Disagree, making the outcomes suitable for logistic regression analyses.

#### Causes and responsibility for chronic disease related factors

Respondents indicated their agreement with items related to alcohol, tobacco, and obesity on a 4-point scale (1 = “Strongly Agree”, 2 = “Agree”, 3 = “Disagree”, and 4 = “Strongly Disagree”). The items were, “When someone has a problem with alcohol it is their own fault”, “When someone has a problem with alcohol it is caused by circumstances beyond their control”, “When someone has a problem with alcohol it is their responsibility to deal with it”, and “When someone has a problem with alcohol it is society’s responsibility to deal with it”. Items were identical except for the subject, which was either alcohol, tobacco, or obesity. Responses to these questions were categorized into Agree and Disagree for analyses.

#### Sociodemographic variables

##### Age

We assessed age by asking, “How old are you today?” Participants then gave their age, which was recorded as a number between 18 and 120. These values were kept continuous for analyses.

##### Educational attainment

Participants were asked, “What is the highest level of education you have attained?” Participants then selected from a list of levels, which were then categorized into either Up to Post-Secondary Education or Post-Secondary Graduate for analyses.

##### Employment status

We assessed employment status by asking, “Which of the following best describes your employment status?” Participants then selected from a list of options, which were then categorized into either Full Time or Other Than Full Time for analyses.

##### Federal or provincial political alignment

We assessed political alignment by asking about federal and provincial election preferences separately. We asked, “If a Federal/Provincial election were held tomorrow, which party’s candidate would you be most likely to support?” Participants then selected from a list of political parties, which were then categorized into Left (New Democratic Party and Green Party), Centre (Liberal Party, Alberta Party), or Right (Conservative Party, Progressive Conservative Party, and Wildrose Party) for analyses.

##### Sex

Sex was assessed by the surveyor who recorded the sex of the participant without asking explicitly. This was recorded as a binary category and treated the same for analyses.

##### Self-reported health status

We assessed self-reported health by asking, “In, general would you say your health is excellent, very good, good, fair or poor?” Participant responses were then categorized into Good or Better (including very good and excellent) or Fair or Worse (including poor) for analyses.

##### Annual household income

We asked, “Which of the following categories best describes the total income of all members of your household for the past year, before taxes and deductions?” Participants then selected from a list of potential income ranges, which were then categorized into Above Median (≥$70,000) or Below Median (<$70,000) for analyses.

##### Number of people in a household

We assessed the number of people in a household by asking, “How many people, including yourself, live in your household?” Participants then gave their response, which was recorded as a number between 1 and 10, and were then categorized into Single, Two, Three, or Four plus Person Households for analyses.

##### Provincial area of residence

We asked participants, “Do you live in the greater Edmonton area, the greater Calgary area, or another city, town or place in Alberta?” Participants then selected from those three options, which were then categorized into Urban (including Edmonton and Calgary) and Rural for analyses.

### Data analysis

#### Missing data and imputation

All data analyses were completed using R version 3.6.0 using the RStudio IDE [21]. Analysis of missing data showed that less than 5% was missing for all variables except for income, and provincial and federal political alignment, for which approximately 30% of observations were missing. Inspection of the data indicated few patterns, Hawkins and non-parametric test of normality and homoscedasticity indicated a rejection of the null hypothesis of data missing completely at random (MCAR) (Hawkins: *p* < .001, Nonparametric: *p* = 0.009). Combined with visual inspection of the data we concluded that the data are likely missing at random (MAR) and thus suitable for multiple imputation [22]. Multiple imputation was done using the multivariate imputation by chained equations method via the *mice* package, using logistic regression to impute binary variables, polynomial regression to impute categorical variables, and predictive mean matching for age. This was completed using 5 iterations and 33 imputations, based on the missingness approaching one third of observations for some variables.

#### Variable selection and modeling

Due to the unique constructs addressed in each question, separate logistic regression models were built for each CDP survey question of interest. Given that the intention was to build explanatory models, models for each question were built by testing of all possible combinations of demographic variables until a stable explanatory model was built that adjusted for any potential confounding between different demographic variables. This effort was also guided in part by a priori knowledge, and by examining the posterior probability that each demographic variable is non-zero from a Bayesian regression process.

Provincial and federal political alignment, in particular, are included in most models together, as a chi-square analysis indicated that most right leaning voters are right leaning both federally and provincially, while the left and centre seem to shift depending on whether they are voting federally or provincially. For most questions we included both, but for some questions they appeared colinear and so only the variable with the higher posterior probability of being non-zero was included. Once a final model was built, it was run on each imputation of the data set, and the coefficients were averaged using Rubin’s rules to generate a final overall output. We then determined odds ratio estimates with 95% confidence intervals for each sociodemographic variable included in the model. Holm’s Sequential Bonferroni Procedure was then used to adjust for familywise error rates for the multiple hypothesis testing in a robust and conservative manner. This procedure is done by ordering all of the tests (for a given set of multiple tests) by *p*-value from smallest to largest, and then testing the smallest probability with the more ubiquitous Bonferroni correction for the number of tests in the given set of multiple tests (where we call the number of tests ‘C’). If the first test comes back as non-significant, no further tests are run. Following a significant test, the second smallest probability is tested in the same way, with a Bonferroni correction for C-1 tests. This process continues until a non-significant result is returned. The corrected *p* value is then calculated for the last test (the *i*th test) as (C-*i* + 1)*p. A more in-depth examination of the procedure can be found in Holm [23]. To assess the validity of the imputation, these same models were run on a complete case version of the original data and the results were compared to assess for difference. The following packages were used to complete the analyses in R: *foreign, stats*, *dplyr*, *tidyr*, *ggplot2*, *GGally*, *ggformula*, *BMA*, *naniar*, *finalfit*, *BaylorEdPsych*, *MissMech*, and *mice*.

## Results

In order to express the results in the most efficient manner, we have presented the variables included in each model, but only the odds ratios and confidence intervals of statistically significant variables after Holm correction in Table 2. For details on each model, please see Additional file 1.

**Table 2:** Logistic Regression on relationships between socio-demographic variables and perceptions of links to cancer, and agreement with statements about cancer and the etiology of chronic disease

**Perceptions of Links to Cancer**	**Model Covariates**	**Significant Variables**	**Odds Ratio (95% Confidence Interval)**
Participating in regular exercise	Age, Education, Sex, Health, Household Income	Education	0.70 (0.55–0.90)
		Sex	0.73 (0.58–0.92)
Maintaining a healthy body weight	Age, Education, Household Income, and Population Centre	Education	0.63 (0.49–0.80)
Smoking cigarettes	Age, Employment, Sex, Health, Household Income, Household Size, and Population Centre	–	–
Using other tobacco products	Age, Education, Employment, Sex, Health, Household Income, and Provincial Vote	–	–
Drinking excessive alcohol	Education, Federal Vote, Sex, Health, Household Income, Population Centre, and Provincial Vote	Sex	0.51 (0.37–0.69)
Where a person works	Age, Education, Employment, Federal Vote, Population Centre, Provincial Vote	–	–
Where a person goes to school	Employment, Sex, Health, and Household income	Sex	0.68 (0.53–0.88)
Eating a healthy balanced diet	Education, Employment, Sex, Health, and Household Income	Household Income	1.54 (1.17–2.03)
Eating sufficient servings of fruits and vegetables	Education, Employment, and Household Income	Education	0.69 (0.55–0.88)
Exposure to tobacco smoke	Education, Federal Vote, Health, and Provincial Vote	–	–
Smoking marijuana	Age, Education, Employment, Federal Vote, Health, Household Income, and Provincial Vote	Age	1.02 (1.01–1.03)
Residing near industrial facilities	Age, Education, Federal Vote, Sex, Household Income, and Provincial Vote	Sex	0.47 (0.31–0.72)
The neighborhood, town or city where a person lives	Age, Education, Employment, Federal Vote, Sex, and Provincial Vote	Sex	0.69 (0.53–0.89)
**Perceptions of Cancer Prevention**	**Model Covariates**	**Significant Variables**	**Odds Ratio (95% Confidence Interval)**
Most cancers are preventable	Age, Education, Sex, Health, and Population Centre	–	–
Cancer prevention only works for young people	Federal Vote, Sex, Household Income, and Provincial Vote	–	–
Cancer is just bad luck; it is not preventable	Age, Education, Employment, Federal Vote, Sex, Household Income, and Provincial Vote	Federal Vote Centre Federal Vote Left Sex	0.26 (0.11–0.61) 0.23 (0.08–0.66) 1.76 (1.28–2.40)
Cancer treatment is more important than prevention	Age, Education, Employment, Federal Vote, Sex, Household Income, and Provincial Vote	Age	1.01 (1.00–1.02)
		Sex	1.52 (1.19–1.95)
Most cancers are caused by genetics	Education, Employment, and Health	–	–
**Perceptions of Causes and Responsibility for Chronic Disease Related Factors**	**Model Covariates**	**Significant Variables**	**Odds Ratio (95% Confidence Interval)**
Alcohol problem - Cause is one’s own fault	Education, Federal, Sex, Household Income, Population Centre, and Provincial Vote	Sex	1.64 (1.29–2.08)
Alcohol problem - Cause is circumstances beyond one’s control	Education, Federal Vote, Health, Household Income, Population Centre, and Provincial Vote	–	–
Alcohol problem – Individual’s responsibility to address	Employment, Federal Vote, Sex, Health, Population Centre, and Provincial Vote	–	–
Alcohol problem – Society’s responsibility to address	Education, Employment, Federal Vote, Household Income, and Provincial Vote	Provincial Vote Left	3.29 (1.69–6.42)
Tobacco problem - Cause is one’s own fault	Education, Federal Vote, Sex, Household Income, and Provincial Vote	Federal Vote Centre	0.47 (0.27–0.83)
		Federal Vote Left	0.29 (0.15–0.57)
Tobacco problem - Cause is circumstances beyond one’s control	Education, Federal Vote, Health, Household Income, and Provincial Vote	Household Income	0.65 (0.47–0.89)
		Provincial Vote Centre	3.67 (1.60–8.39)
Tobacco problem - Individual’s responsibility to address	Education, Federal Vote, Sex, Health, Population Centre, and Provincial Vote	Federal Vote Left	0.27 (0.11–0.64)
Tobacco problem - Society’s responsibility	Education, Federal Vote, and Provincial Vote	–	–
Obesity problem - Cause is one’s own fault	Age, Federal Vote, Sex, Household Size, and Provincial Vote	Age	0.99 (0.98–0.99)
		Federal Vote Left	0.46 (0.24–0.88)
		Household Size 4+ Persons	0.57 (0.39–0.83)
Obesity problem - Cause is circumstances beyond one’s control	Federal Vote, Health, Household Income, Household Size, and Provincial Vote	–	–
Obesity problem - Individual’s responsibility to address	Age, Employment, Federal Vote, Health, and Provincial Vote	Federal Vote Left	0.33 (0.15–0.73)
Obesity problem - Society’s responsibility	Education, Federal Vote, and Provincial Vote	Education	0.66 (0.49–0.88)

Note. For perceptions of links to cancer, participants were asked, “Please indicate how much you think each of the following items is linked to a person’s chances of getting cancer” which was dichotomized into “linked/not linked”. For statements about cancer and the etiology of chronic disease, participants were asked, “Please indicate your level of agreement with each of the following statements” which was dichotomized into “agree/disagree”

### Behavioural and environmental links to cancer

For questions on how much a given behaviour or environment is linked to cancer, the given odds ratios correspond to the odds of there being a perceived link. The details on model covariates and odds ratios for these questions can be found in Table 2. Men (compared to women, all else being equal) were less likely to link regular exercise (OR: 0.73, 95CI: 0.58–0.92), or drinking excessive alcohol (OR: 0.51, 95CI: 0.37–0.69), to reducing or increasing cancer risk, respectively. Men were also less likely to link where a person goes to school (OR: 0.68, 95CI: 0.53–0.88), residing near industrial facilities (OR: 0.47, 95CI: 0.31–0.72), or the neighbourhood, city, or town where a person lives (OR: 0.69, 95CI: 0.53–0.89) to a person’s cancer risk. Older people were more likely to link smoking marijuana (OR: 1.02, 95CI: 1.01–1.03) with cancer compared to younger people. Those with less than post-secondary education (compared to their more educated peers) were less likely to believe that regular exercise (OR: 0.70, 95CI: 0.55–0.90), maintaining a healthy body weight (OR: 0.63, 95CI: 0.49–0.80), or eating sufficient fruits and vegetables (0.69, 95CI: 0.55–0.88) were linked to cancer. Households making above the median income were more likely to link eating a balanced diet with cancer compared to those making below the median income (OR: 1.54, 95CI: 1.17–2.03), all else being equal.

### Cancer prevention

For questions measuring agreement with statements about cancer prevention, the odds ratios here correspond to the odds of agreement. The details on model covariates and odds ratios for these questions can be found in Table 2. Men (compared to women) were more likely to agree that cancer was not preventable (OR: 1.76, 95CI: 1.28–2.40), and that cancer treatment is more important than prevention (1.52, 95CI: 1.19–1.95). Older people were also more likely to believe that cancer treatment is more important than prevention, compared to younger people (OR: 1.01, 95CI: 1.00–1.02). Left- (OR: 0.26, 95CI: 0.11–0.61) and centre-leaning (OR: 0.23, 95CI: 0.08–0.66) federal voters were more likely to believe that cancer is just bad luck and not preventable compared to right-leaning voters.

### Causes and responsibility

For questions measuring agreement with statements about the etiology of, and responsibility for, chronic disease, the odds ratios here correspond to the odds of agreement. The details on model covariates and odds ratios for these questions can be found in Table 2. Men were more likely to believe that alcohol problems are the fault of the individual compared to women (OR: 1.64, 95CI: 1.29–2.08). Older people were less likely to believe obesity is one’s own fault compared to younger respondents (OR: 0.99, 95CI: 0.98–0.99). Left-leaning provincial voters were more likely to believe that alcohol problems are society’s responsibility (OR: 3.29, 95CI: 1.69–6.42), and left-leaning (OR: 0.47, 95CI: 0.27–0.83) and central federal voters (OR: 0.29, 95CI: 0.15–0.57) were less likely to believe tobacco problems are one’s own responsibility to address, compared to their right-leaning counterparts. Central provincial voters were more likely to believe that tobacco problems were caused by problems outside of one’s control (OR: 3.67, 95CI: 1.60–8.39) (compared to right-leaning). Left-leaning federal voters (relative to right-leaning voters) were less likely to think that tobacco (OR: 0.27, 95CI: 0.11–0.64) or obesity problems (OR: 0.33, 95CI: 0.15–0.73) should be addressed individually. For obesity, left (vs. right) federal voters were less likely to believe that obesity is caused by one’s own fault (OR: 0.46, 95CI: 0.24–0.88) and that obesity is an individual’s responsibility to address (OR: 0.33, 95CI: 0.15–0.73). Those with less education were less likely to believe that society is responsible for addressing obesity compared to more educated respondents (OR: 0.66, 95CI: 0.49–0.88). Those with higher incomes were less likely to think that tobacco problems were caused by circumstances beyond one’s control compared to those with lower incomes (OR: 0.65, 95CI: 0.47–0.89). Those in large households (4 or more persons) were less likely to believe that obesity is one’s own fault compared to those in smaller households (OR: 0.57, 95CI: 0.39–0.83).

## Discussion

The results of this analysis demonstrate several patterns in the socio-demographic factors related to knowledge and beliefs about cancer and cancer prevention as well as causes and responsibility for alcohol use, tobacco use, and obesity. The patterns have implications for message framing, knowledge sharing, and policy.

We hypothesised that women (compared to men) would recognize environmental factors related to cancer, and social responsibility for alcohol, tobacco, and obesity. Our findings that men were less likely to link several individual or environmental factors to cancer risk or prevention compared to women are in line with research showing women were more aware of social determinants of health [11]. In Canada, men were more likely than women to be smokers, heavy drinkers, and to be classified as obese [24]. Men, however, were more likely to do regular physical activity compared to women [25]. Men’s health behaviour and beliefs about health risk and responsibility may be partially determined by societal constructions of masculinity. In general, men are less likely than women to engage in health promoting behaviour, and many researchers have identified traditional conceptualizations of masculinity as contributing to men’s health behaviour that would need to be rejected to enact positive health behaviours (e.g., men are invulnerable to risks associated with unhealthy behaviour, not be interested in learning about health, and would not be concerned about weight, diet, or hygiene) [26–30]. Men’s enacting of sex and gender roles and perceptions of invulnerability may partially explain the lack of acknowledgement in this sample of the environmental or societal influences on chronic disease or its determinants.

In our examination of age, we found that older people were more likely to link smoking marijuana with cancer and were more likely to believe that cancer treatment is more important than prevention than were younger people. In agreement with our hypotheses, older people were less likely to believe obesity is one’s own fault. In other research, younger age was associated with knowledge of actual and mythical causes of cancer [31]. In contrast to our findings about cancer treatment and prevention, age has been positively associated with acknowledgement of social determinants of health [11], support for nutrition policies [32] and more intrusive interventions, which may indicate a greater awareness of chronic disease burden or trust in the government [12]. Older populations may be useful in supporting prevention-focused health campaigns and providing mentorship for younger people, but this may depend on the target of prevention efforts.

Aligned with our expectations for political orientation, those who were left-leaning or central federal voters were less likely to believe that cancer cannot be prevented compared to right-leaning federal voters. For health behaviours, left-leaning and central voters were more likely to support societal responsibility and responsibility for alcohol, tobacco, and obesity compared to right-leaning voters.

Alberta has elected conservative provincial leaders (right-leaning) for over four decades. The only successful left-leaning party elected in recent history was the New Democratic Party (NDP) who were elected as a majority provincial government from 2015 to 2019. Alberta’s NDP party represents a more central political mandate compared to their federal counterparts. This alignment may explain the overlap between central federal and left provincial voting patterns and beliefs. Typically, left-leaning voters are more open to policies such as taxation, advertising bans, and city planning initiatives to create healthier environments theoretically due to placing a high value on the positive effect our social environments can have on our health [13]. Understanding a person’s alignment in their provincial and federal voting patterns may provide more insight into the types of policies they may be likely to support.

Components of socioeconomic status appeared to play important roles in perceptions of cancer and chronic disease correlates. Those with less than post-secondary education were less likely to link exercise, body weight, or fruit and vegetable intake to cancer, but there were no effects for environmental causes compared to those with more education. Those with less education were also less likely to believe that society is responsible for addressing obesity. Our results are in line with findings that underserved populations generally had less knowledge of cancer risk factors [31], but contradicts research that identified that those with less education were more likely to recognize social determinants of health [11]. Similarly, those reporting a more affluent household were more likely to link eating a balanced diet with cancer compared to those from less affluent households. In addition, those with higher incomes were less likely to think that tobacco problems were caused by uncontrollable circumstances. These results partially support our hypotheses and, unlike our findings for education, the results are consistent with research showing that those who recognized social determinants of health were more likely to have less income and poorer health [11]. These findings highlight the intersections of education, income, and health, which are social determinants instrumental in mitigating structural barriers to health and well-being. Those with more income may be less aware of the importance of one’s life circumstances in their health behaviour and thus attribute personal responsibility for these actions, but these relationships may be moderated by factors like education and health status.

This study provides insight into the importance of health literacy, message framing, and potential of socio-demographic factors to impact healthy policy. Health literacy has been linked with supporting nutrition policies in young Canadians [32]. Health literacy and understanding accurate risk for cancer and other chronic diseases can reduce fear and worry, and empower people to reduce their risk [31]. Unfortunately, poor health literacy was found among men, racial/ethnic minorities, those who were unable to work, those with stronger religious beliefs, and those with higher perceived social influence among adults aged 50–70 accessing primary care services [33]. Our research supports and extends this research showing that men, those with less education, and those with less income may have poorer health literacy. Targeting these socio-demographic groups may be important priorities for improving health behaviour and support for healthy policy, particularly through upstream interventions.

Several researchers have identified strategies informed by attribution theory to communicate health risk information to promote understanding of the social determinants of health. Researchers have suggested that we must still acknowledge the role of personal responsibility in health, but also explicitly highlight the incremental changes that can be supported through policy to improve community health without imposing on individual freedoms [5, 11, 34, 35]. Increasing the salience of upstream causes of obesity and other health conditions may result in individuals who were more likely to hold governments and corporations accountable, which can motivate collective action aimed at changing policy and improving the well-being of their communities [34]. Another strategy stemming from attribution theory is to assign agency to the disease or risk factor (e.g., obesity causes health problems) rather than the individual (e.g., obese people experience health problems) to promote environmental or genetic causes of a condition as explored in research by McGlynn and McGlone [36]. Our models suggest that those with more income, men, those with less education, and right-leaning voters may benefit from improved messaging around causes and responsibility for chronic disease beyond those messages that emphasize personal agency. Communications about health behaviour and healthy public policy should integrate behaviour-specific messaging research to avoid stigmatizing people and support upstream approaches to health promotion.

### Limitations

The present analyses were not without limitation. We did not measure if a person is living with a chronic disease, which may be related to their perceptions of chronic disease prevention and cause [37]. We were unable to address race or culture in these analyses as this information was not collected from participants, which is unfortunate as it has been shown to influence perceptions of chronic disease [11]. Further, the determination of sex based on interviewer impression may have led to inaccuracies in recording of sex, and did not adequately account for the socio-cultural continuum of gender. Non-response and “don’t know” responses were pooled in the final data, inadequately accounting for respondents with no opinion (both have been rectified in later iterations of the CDP survey). The models used here are also relatively simple and did not look at the potential for interaction terms, which may impact the accuracy of the estimates of a causal odds ratio. This analysis was, however, largely exploratory, and the complexity of interpretation for these terms were out of scope.

The moderate amount of missing data (including “don’t know” responses), largely concentrated among questions pertaining to household income and political alignment, was a limitation in that it impacted the sample size and power of analyses when working with complete cases. We addressed this, however, by using multiple imputation to improve the power and precision of this analysis. Multiple imputation here assumes that the data is missing at random, and not dependant on unobserved data. While impossible to verify statistically, the statistically important differences in demography between those who answered a question versus those who did not, and the agreement between the coefficients of the complete case and imputed and pooled models support that this is a reasonable assumption.

## Conclusions

The present analyses modelled relationships between important socio-demographic factors and beliefs about causes, risks, and responsibility for chronic disease and its risk factors. The results demonstrate the importance of targeting subgroups to bring awareness to when providing information or advocating for healthy public policy. Researchers can extend this work by identifying and examining specific interactions between socio-demographic variables in predicting chronic disease and policy perspectives. Furthermore, it is critical to identify mechanisms by which these socio-demographic variables work to generate critical beliefs and perspectives which inform policy support.

## Supplementary Information

**Additional file 1.** An additional table is available describing the variables and regression results included in each binary logistic regression model created for each survey question separated by imputed and complete case datasets. The file name is “BLR-Additional Table 1-BMC.xlsx” and it titled “Logistic Regression Models”.

## Data Availability

The datasets generated and/or analysed during the current study are not publicly available due to requirements of our research ethics approval. Data from the 2016 Chronic Disease Prevention Survey are available from the corresponding author on reasonable request.

## Abbreviations

NCD: Noncommunicable disease
CDP: Chronic Disease Prevention (survey)

## References

[1] World Health Organization. Noncommunicable diseases. 2018. https://www.who.int/news-room/fact-sheets/detail/noncommunicable-diseases. Accessed 15 Oct 2019.

[2] Marmot M, Friel S, Bell R, Houweling TAJ, Taylor S (2008). Closing the gap in a generation: health equity through action on the social determinants of health. Lancet.

[3] CSDH (2008). Closing the gap in a generation: health equity through action on the social determinants of health. Final report of the commission on social determinants of health.

[4] Magnusson RS (2015). Case studies in nanny state name-calling: what can we learn?. Public Health.

[5] Lundell H, Niederdeppe J, Clarke C (2013). Public views about health causation, attributions of responsibility, and inequality. J Health Commun.

[6] McLeroy KR, Bibeau D, Steckler A, Glanz K (1998). An ecological perspective on health promotion programs. Health Educ Q.

[7] Yun L, Vanderloo LM, Berry TR, Latimer-Cheung AE, O’Reilly N, Rhodes RE, et al. Political orientation and public attributions for the causes and solutions of physical inactivity in Canada: Implications for policy support. Front Public Health. 2019;7:153. 10.3389/fpubh.2019.00153 PMC6611409 31316958

[8] Weiner B (1985). An attributional theory of achievement motivation and emotion. Psychol Rev.

[9] Weiner B (1993). On sin versus sickness: a theory of perceived responsibility and social motivation. Am Psychol.

[10] Weiner B (1991). Metaphors in motivation and attribution. Am Psychol.

[11] Robert SA, Booske BC (2011). US opinions on health determinants and social policy as health policy. Am J Public Health.

[12] Furnham A (1994). Explaining health and illness: lay perceptions on current and future health, the causes of illness, and the nature of recovery. Soc Sci Med.

[13] Niederdeppe J, Robert SA, Kindig DA. Qualitative research about attributions, narratives, and support for obesity policy, 2008. Prev Chronic Dis. 2011;8(2):39. PMC3073432 21324253

[14] Diepeveen S, Ling T, Suhrcke M, Roland M, Marteau TM (2013). Public acceptability of government intervention to change health-related behaviours: a systematic review and narrative synthesis. BMC Public Health.

[15] Boiarsky G, Rouner D, Long M (2013). Effects of responsibility attribution and message source on young adults’ health attitudes and behaviors. J Health Commun.

[16] Raine KD, Nykiforuk CIJ, Vu-Nguyen K, Nieuwendyk LM, VanSpronsen E, Reed S, Wild TC (2014). Understanding key influencers’ attitudes and beliefs about healthy public policy change for obesity prevention. Obesity..

[17] Haley H, Sidanius J (2006). The positive and negative framing of affirmative action: a group dominance perspective. Personal Soc Psychol Bull.

[18] Zuck N (2000). Decision latitude, self-determination, and participation in workplace health promotion programs.

[19] Cohen JE, de Guia NA, Ashley MJ, Ferrence R, Northrup DA, Studlar DT (2002). Predictors of Canadian legislators’ support for tobacco control policies. Soc Sci Med.

[20] Nykiforuk CIJ, Wild TC, Raine KD (2014). Cancer beliefs and prevention policies: comparing Canadian decision-maker and general population views. Cancer Causes Control.

[21] R Core Team (2017). R: A language and environment for statistical computing.

[22] Donders ART, Heijden, van der G. GJMG, Stijnen T, Moons KGM. Review: a gentle introduction to imputation of missing values. J Clin Epidemiol 2006;59:1087–1091. doi:10.1016/j.jclinepi.2006.01.014, 10. 10.1016/j.jclinepi.2006.01.014 16980149

[23] Holm S. A simple sequentially rejective multiple test procedure. Scand J Stat. 1979;6(2):65-70.

[24] Statistics Canada. The daily — Canadian community health survey, 2018. 2019. https://www.150.statcan.gc.ca/n1/daily-quotidien/190625/dq190625b-eng.htm. Accessed 29 Mar 2020.

[25] Statistics Canada. The daily — Canadian health measures survey: directly measured physical activity of Canadians, 2012 and 2013. 2015. http://www.statcan.gc.ca/daily-quotidien/150218/dq150218c-eng.htm. Accessed 23 Aug 2015.

[26] Sloan C, Conner M, Gough B (2015). How does masculinity impact on health? A quantitative study of masculinity and health behavior in a sample of UK men and women. Psychol Men Masculinity.

[27] Dawson KA, Schneider MA, Fletcher PC, Bryden PJ (2007). Examining gender differences in the health behaviors of Canadian university students. J R Soc Promot Heal.

[28] Courtenay WH (2000). Constructions of masculinity and their influence on men’s well-being: a theory of gender and health. Soc Sci Med.

[29] Courtenay WH (2000). Engendering health: a social constructionist examination of men’s health beliefs and behaviors. Psychol Men Masculinity.

[30] Mahalik JR, Levi-Minzi M, Walker G (2007). Masculinity and health behaviors in Australian men. Psychol Men Masculinity..

[31] Shahab L, McGowan JA, Waller J, Smith SG (2018). Prevalence of beliefs about actual and mythical causes of cancer and their association with socio-demographic and health-related characteristics: findings from a cross-sectional survey in England. Eur J Cancer.

[32] Bhawra J, Reid JL, White CM, Vanderlee L, Raine K, Hammond D (2018). Are young Canadians supportive of proposed nutrition policies and regulations? An overview of policy support and the impact of socio-demographic factors on public opinion. Can J Public Health.

[33] Christy SM, Gwede CK, Sutton SK, Chavarria E, Davis SN, Abdulla R, Ravindra C, Schultz I, Roetzheim R, Meade CD (2017). Health literacy among medically underserved: the role of demographic factors, social influence, and religious beliefs. J Health Commun.

[34] Sun Y, Krakow M, John KK, Liu M, Weaver J (2016). Framing obesity: how news frames shape attributions and behavioral responses. J Health Commun.

[35] Brownell KD, Kersh R, Ludwig DS, Post RC, Puhl RM, Schwartz MB, Willett WC (2010). Personal responsibility and obesity: a constructive approach to a controversial issue. Health Aff.

[36] McGlynn J, McGlone MS (2019). Desire or disease? Framing obesity to influence attributions of responsibility and policy support. Health Commun.

[37] French DP, Senior V, Weinman J, Marteau TM (2001). Causal attributions for heart disease: a systematic review. Psychol Health.

